# Comparing mortality between positive and negative blood culture results: an inverse probability of treatment weighting analysis of a multicenter cohort

**DOI:** 10.1186/s12879-021-05862-w

**Published:** 2021-02-17

**Authors:** Aibo Liu, Chia-Hung Yo, Lu Nie, Hua Yu, Kuihai Wu, Hoi Sin Tong, Tzu-Chun Hsu, Wan-Ting Hsu, Chien-Chang Lee

**Affiliations:** 1 Department of Laboratory Medicine, Sichuan Provincial People’s Hospital University of Electronic Science and Technology of China, Chengdu, China; 2 Chinese Academy of Sciences Sichuan Translational Medicine Research Hospital , Chengdu, China; 3 Department of Emergency Medicine, Far Eastern Memorial Hospital, Taipei, Taiwan; 4 Department of Laboratory Medicine, The First People’s Hospital of Foshan, Foshan, Guangdong China; 5 The University of Hong Kong, Hong Kong, China; 6 Department of Emergency Medicine, National Taiwan University Hospital, Taipei, Taiwan; 7 Department of Epidemiology, Harvard TH Chan School of Public Health, Boston, MA USA; 8 Health Data Science Research Group, National Taiwan University Hospital, No. 7, Chung-Shan South Road, Taipei, 100 Taiwan; 9 The Centre for Intelligent Healthcare, National Taiwan University Hospital, No. 7, Chung-Shan South Road, Taipei, 100 Taiwan

**Keywords:** Inverse probability of treatment weighting, Sepsis, *Staphylococcus aureus*, Mortality

## Abstract

**Background:**

The association between blood culture status and mortality among sepsis patients remains controversial hence we conducted a tri-center retrospective cohort study to compare the early and late mortality of culture-negative versus culture-positive sepsis using the inverse probability of treatment weighting (IPTW) method.

**Methods:**

Adult patients with suspected sepsis who completed the blood culture and procalcitonin tests in the emergency department or hospital floor were eligible for inclusion. Early mortality was defined as 30-day mortality, and late mortality was defined as 30- to 90-day mortality. IPTW was calculated from propensity score and was employed to create two equal-sized hypothetical cohorts with similar covariates for outcome comparison.

**Results:**

A total of 1405 patients met the inclusion criteria, of which 216 (15.4%) yielded positive culture results and 46 (21.3%) died before hospital discharge. The propensity score model showed that diabetes mellitus, urinary tract infection, and hepatobiliary infection were independently associated with positive blood culture results. There was no significant difference in early mortality between patients with positive or negative blood culture results. However, culture-positive patients had increased late mortality as compared with culture-negative patients in the full cohort (IPTW-OR, 1.95, 95%CI: 1.14–3.32) and in patients with severe sepsis or septic shock (IPTW-OR, 1.92, 95%CI: 1.10–3.33). After excluding Staphylococcal bacteremia patients, late mortality difference became nonsignificant (IPTW-OR, 1.78, 95%CI: 0.87–3.62).

**Conclusions:**

Culture-positive sepsis patients had comparable early mortality but worse late mortality than culture-negative sepsis patients in this cohort. Persistent Staphylococcal bacteremia may have contributed to the increased late mortality.

**Supplementary Information:**

The online version contains supplementary material available at 10.1186/s12879-021-05862-w.

## Background

Sepsis is a leading cause of morbidity and mortality among hospitalized patients and results in a significant healthcare burden [1]. In the United States, severe sepsis and septic shock remain the dominant cause of death in intensive care units (ICUs), with approximately 900,000–3,000,000 cases yearly [2]. Economically, sepsis was ranked in the top four most expensive conditions, costing an aggregate of $20,298,000 yearly in US hospitals [3, 4]. Mortality from severe sepsis and septic shock remains unacceptably high, ranging between 20 and 40% depending on the severity of illness [5]. Blood culture remains an essential test in the care of sepsis patients [6]. The results of blood culture can be used to guide the adjustment of empiric antibiotics regimen [7]. Blood culture positivity has been suggested as a surrogate marker of bacterial load, as evidenced by the worse prognosis in sepsis patients with a shorter time from blood culture incubation to growth detection [8]. However, isolation of specific microorganisms by blood culture remains challenging [9]. In approximately one third to two thirds of sepsis patients, no specific organism can be identified by culture [10].

Culture-positive patients were thought to have a higher burden of infection and therefore worse outcomes [11, 12]. However, contrary to the general belief, recent studies have shown that culture-negative and culture-positive sepsis patients are generally comparable in resource utilization and outcomes [13, 14]. To date, only a few studies have addressed the epidemiology and outcomes of culture-negative sepsis [2, 10]. Several questions remain to be answered. First, previous studies typically included patients with severe sepsis or septic shock rather than the whole spectrum of sepsis [2, 14]. Second, previous studies only focused on in-hospital or 30-day mortality.

We therefore extended the analysis to include the whole spectrum of Systemic inflammatory response syndrome (SIRS)-defined sepsis patients and followed the patients until 90 days to see if blood culture positivity may affect the mid-term outcome in sepsis patients.

## Methods

### Patient population

The investigation was a retrospective multicenter cohort study. During a 24-month period from 1 January 2015 through 31 December 2016, the charts of all patients who were admitted to the emergency department, hospital floor, or ICU with a presumed systemic infection and who received blood cultures were reviewed. We included patients who fulfilled the criteria of systemic inflammatory syndrome and excluded patients with missing data or do-not-resuscitate (DNR) orders. We also excluded patients transferred from other hospitals or patients lost to follow-up.

### Collection of clinical variables

To verify the presence of infection as a cause for admission, participating investigators from the three locations independently examined all the collected medical records. The clinical and laboratory data were collected at the time of admission. The definition of the variable collected has been described in our previous work. Briefly, we defined SIRS as two of the following: temperature > 38 °C or < 36 °C, heart rate > 90 beats/min, respiratory rate > 20 breaths/min or PaCO_2_ < 32 mmHg (4.3 kPa), white cell count > 12,000 cells/μl or < 4000 cells/μl, and 10% immature/band forms. We defined severe sepsis as sepsis associated with organ dysfunction and septic shock as having a systolic blood pressure <  90 mmHg requiring vasopressor therapy. Organ dysfunctions assessed at admission were based on sequential organ failure assessment (SOFA) score with modifications for use outside an ICU setting, which are defined as follows: 1) altered consciousness, a Glasgow Coma Scale score of < 14 or a decrease in the score of at least 2 if a primary central nervous system injury was present, 2) acute respiratory failure, a pulse oxygen saturation <  90% at admission or requirement of mechanical ventilation, 3) acute kidney injury, a serum creatinine level > 2.0 mg/dL or, in the case of preexisting renal dysfunction, a doubling of previous serum creatinine values, 4) acute hepatic dysfunction, an increase in bilirubin concentration > 2 mg/dL and the occurrence of coagulation disorders with INR > 1.5, 5) acute hematologic dysfunction, an decrease in platelet count < 100 × 10^3^/μL, and 6) acute cardiovascular dysfunction, the presence of hypotension requiring vasopressor. We also applied quick sequential organ failure assessment (quick SOFA) for patient severity classification [15]. The criteria for quick SOFA include altered level of consciousness (Glasgow coma score < 14), hypotension (blood pressure < 100 mmHg), or tachypnea (> 22 breaths per min). In patients with dementia or delirium, altered mental status was used instead of GCS.

The conditions of the patients were being recorded for at least 90 days, which includes the duration of hospital stay and clinic appointments after discharge. For all eligible patients, the following data were collected using a standard case report form: demographic features, the presenting co-morbid medical states, initial vital signs, requirements for assisted ventilation or hemodialysis, diagnosis during admission and discharge, and the identity of blood-isolated microorganisms. In the final discharge diagnosis, the occurrence and origins of a focal infection was categorized as lower respiratory tract infection, hepatobiliary tract infection, urinary tract infection, soft tissue and skin infection, intra-abdominal infection, and other infections. We reviewed past medical records to collect the comorbidity information. Diabetes mellitus was defined by discharge diagnosis with use of hypoglycemic agents or insulin. Chronic pulmonary disease include chronic obstructive pulmonary disease (COPD), asthma, bronchiectasis, and chronic bronchitis. Hemiplegic stroke was defined as stroke with hemiplegia or being wheelchair bound. Previous myocardial infarction and congestive heart failure was defined by discharge diagnosis and use of related medications. Chronic liver disease includes chronic viral hepatitis or liver cirrhosis. Chronic kidney disease includes stage four or stage five diseases. Malignancies include both solid and hematologic cancer.

### Blood culture and determination of bloodstream infection

During the study period, anaerobic and aerobic blood cultures were routinely used for patients with presumed systemic infection. Blood cultures were performed using the BacT/ALERT FA and BacT/ALERT (bioMérieux, Marcy-l’Etoile, France) bottles. Each bottle was incubated into the BacT/ALERT 3D instrument until signs of positive culture or a maximum of 5 days have passed. All positive bottles (including both aerobic and anaerobic BCs) were systematically plated on blood agar plate (BAP)/eosin methylene blue (EMB) agar, chocolate and anaerobic blood agar and incubated under anaerobic (10%CO2 + 5%H2 + 85%N2; Concept 400, Ruskinn Technology, Guiseley, UK) and aerobic conditions. All isolated were identified from the colonies using a standard algorithm using growth on differential agar and biochemical tests, followed by definitive identification with Phoenix 100 (Becton Dickinson, Franklin Lakes, New Jersey, USA). Clinically significant bloodstream infection (BSI) was defined as at least two sets of positive blood cultures from separate sites, one set positive for a Gram-negative bacterial pathogen or one set positive for a Gram-positive pathogen in a patient with an intravascular device and clinical compatibility. It was deemed as a true infection if the microorganisms isolated from the blood cultures included *Staphylococcus aureus*, *Streptococcus pyogenes, Streptococcus agalactiae, Streptococcus pneumoniae, Escherichia coli* and other members of the family Enterobacteriaceae, *Pseudomonas aeruginosa,* the *Bacteroides fragilis* group, and Candida species. Contamination was presumed if blood cultures were positive for *Propionibacterium acnes*, Corynebacterium spp., or Bacillus spp. In addition, when Coagulase-negative staphylococcus was isolated from only one of at least two sets of blood cultures, it was also regarded as contamination. Primary BSI was defined as a laboratory-confirmed BSI that was not secondary to an infection at another body site, whereas secondary BSI was designated as BSI attributable to a primary site of infection. This study was approved by the Research Committees and Institutional Review Boards for all institutions, and it met the criteria for exemption from informed consent.

### Statistical analysis

Absolute counts with relative frequencies were reported for categorical variables, and the median with 25th and 75th percentiles was reported for continuous variables. Univariate comparison was performed with Chi-square tests for categorical variables and Mann-Whitney U tests for continuous variables. We evaluated the impact of blood culture positivity on the early and late mortality of sepsis. Given known differences in patient characteristics between blood culture-positive and blood culture-negative patients, inverse probability of treatment weighting (IPTW) was employed to control confounding caused by baseline general health disparities in the two groups of patients [16–18].

The IPTW approach weights individuals based on their propensity score, with those patients with a high likelihood of positive culture weighted lower; its goal is to create weighted individual samples where the measured baseline covariates match between the two groups [19, 20]. In this study, the PS was defined as the conditional probability of culture-positive compared to culture-negative results for the index episode of sepsis. By creating a logistic regression model with 19 covariates representing patient demographics and features that are proven to be related to blood culture positivity and the results, we estimated a PS for blood culture-positive sepsis. For the model of PS component variables and their respective weights, see Supplementary Table 2. To visualize the balance of covariates after PS matching, we plotted the standardized mean differences of all covariates (Supplementary Figure 2). We used the IPTW to evaluate the logistic regression model in the IPTW weighting study and obtained an estimation that is independent of the possible confounding of all component variables in the PS. The IPTW-weighting study used robust sandwich variance estimators to establish a reliable confidence interval. All tests were 2- tailed, and *p* values < 0.05 were considered statistically significant. The 90-day survival curve was plotted, and the cumulative survival between culture-positive and culture-negative sepsis patients was compared with the log rank test. All analyses were conducted using SAS Version 9.4 (SAS Inc., Cary, NC, USA). A 2-sided *p*-value < 0.05 was viewed as significant.

## Results

### Study cohort and patient characteristics

In this tri-center cohort, 1405 patients with SIRS-defined sepsis were identified, comprising 655 (46.6%) patients with severe sepsis and 272 (19.4%) patients with septic shock. Of the total patients, 1189 (85.9%) were identified as culture-negative sepsis patients and 216 (15.4%) as culture-positive sepsis patients. There were no gender differences between the culture-positive and culture-negative sepsis patients. The culture-positive patients were slightly older and more likely to have diabetes mellitus and chronic kidney disease than their culture-negative sepsis counterparts. When evaluating the source of infection, patients with urinary tract infection or hepatobiliary tract infection were more likely to have positive blood culture results, while patients with lower respiratory tract infection (LRTI) were more likely to have negative blood culture results (Table 1). A flowchart of the patient selection process is shown in Supplementary Figure 1. We provided the information on the microbiology of bacteria isolated in Supplementary Table 3.

**Table 1:** Characteristics of encounters with suspected Infection and fulfilled the SIRS criteria at 3 participating hospitals from 2015 to 2016 (*N* = 1405)

	Culture negative (***n*** = 1189)	Culture positive (***n*** = 216)	***P*** value
**Demographic**			
Sex (Male %)	745 (62.7%)	137 (63.4%)	0.830
Age, median (interquartile range)	62.0 (47.0, 75.0)	68.0 (57.0, 76.8)	0.001*
Age, year > = 65	551 (46.3%)	126 (58.3%)	0.0012*
Nursing home residents	61 (5.1%)	14 (6.5%)	0.4164
**Comorbidities**			
Diabetes mellitus	248 (20.9%)	77 (35.6%)	<.0001*
Chronic pulmonary disease	71 (6.3%)	16 (7.8%)	0.399
Hemiplegic stroke	160 (13.5%)	32 (14.8%)	0.593
Previous myocardial infarction	36 (3.0%)	7 (3.2%)	0.867
Congestive heart failure	80 (6.7%)	22 (10.2%)	0.072*
Chronic liver disease	109 (9.2%)	29 (13.4%)	0.053
Chronic kidney disease	99 (8.3%)	30 (13.9%)	0.009*
Malignancies	120 (10.1%)	32 (14.8%)	0.040
**Site of primary infection**			
Lower respiratory tract infection	664 (55.8%)	104 (48.1%)	0.037*
Urinary tract infection	120 (10.1%)	39 (18.1%)	0.001*
skin and soft tissue infection	33 (2.8%)	8 (3.70%)	0.456
Hepatobiliary infection	48 (4.0%)	22 (10.2%)	< 0.001*
Intra-abdomen infection	80 (6.7%)	15 (6.9%)	0.907

*p < 0.05

### Clinical characteristics and outcomes

We compared the presenting vital signs, laboratory markers, organ dysfunction, and clinical severity scores between culture-positive and culture-negative sepsis patients. Culture-positive sepsis patients were more likely to develop septic shock, high fever, leukocytosis, thrombocytopenia, procalcitoninemia, and lactatemia. Culture-positive sepsis patients were more likely to develop acute organ dysfunction, except for acute respiratory failure. Severity of illness, measured as the proportion of patients with a quick SOFA score greater than 2, was similar between culture-positive and culture-negative sepsis, whereas culture-positive sepsis patients had higher MEDS scores (Mortality in Emergency Department Sepsis Score). Median hospital stay was longer in the culture-positive group (15 days vs. 13 days, *p* = 0.003). The 90-day mortality was significantly higher in the culture-positive sepsis patients (21.3% vs. 12.9%, *p* = 0.001), but 30-day mortality was comparable in both groups (8.8% vs. 8.2%) (Table 2).

**Table 2:** Comparison of clinical and laboratory characteristics collected at the time of admission between culture negative and culture positive patients

	Culture negative (***n*** = 1189)	Culture positive (***n*** = 216)	***P*** value
**Vital signs**			
Systolic blood pressure (mmHg)	122 (108,138)	124 (108,143)	0.397
Shock (< 90 mmHg)	215 (18.1%)	55 (25.5%)	0.011*
Pulse rate	100 (85,113)	102 (86,115)	0.165
Tachycardia (PR > 90/ min)	777 (65.3%)	145 (67.1%)	0.612
Body temperature	37.2 (36.6, 38.3)	37.5 (36.7, 38.7)	0.024*
Fever > 38 °C	405 (34.1%)	91 (42.1%)	0.022*
Hypothermia < 36 °C	22 (1.9%)	9 (4.2%)	0.033*
**Laboratory markers**			
WBC count (1000/mm3)	9630 (6092,12,865)	10,760 (716,16,530)	0.003*
Leukocytosis (WBC > 12,000/mm3)	388 (32.6%)	94 (43.5%)	0.02*
Leukopenia (WBC < 4000/mm3)	124 (10.4%)	16 (7.4%)	0.173
Platelet count (1000/mm3)	178 (107–256)	153 (102–223)	0.02*
Thrombocytopenia (< 150,000/mm3)	479 (40.3%)	105 (48.6%)	0.022*
Procalcitonin	0.74 (0.16, 5.07)	1.34 (0.30, 10.74)	< 0.001*
C-reactive protein	76.1 (28.5, 138.8)	72.1 (19.4, 162.8)	0.572
Lactate	1.8 (1.20–3.07)	2.1 (1.5, 4.4)	0.003*
**Organ dysfunction**			
Altered consciousness	199 (16.17%)	48 (22.2%)	0.051*
Acute respiratory failure	266 (22.4%)	58 (26.9%)	0.150
Acute kidney injury	172 (14.5%)	49 (22.7%)	0.002*
Acute hepatic dysfunction	232 (19.5%)	57 (26.4%)	0.021*
Acute hematologic dysfunction	479 (40.3%)	105 (48.6%)	0.022*
Acute cardiovascular dysfunction	215 (18.1%)	55 (25.5%)	0.011*
**Severity score**			
quick SOFA criteria > = 2	191 (16.1%)	43 (19.9%)	0.163
MEDS score	6 (3,9)	8 (5,11)	< 0.001
**Length of stay**			
Hospital	13 (7, 21)	15 (8, 26)	0.003
**Mortality**			
90-day mortality	153 (12.9%)	46 (21.3%)	0.001
Early (30-day) mortality	98 (8.2%)	19 (8.8%)	0.786
Late (30–90 day) mortality	55 (4.6%)	27 (12.5%)	< 0.001

*p < 0.05

### IPTW weighted outcome comparison

**Fig. 1:**
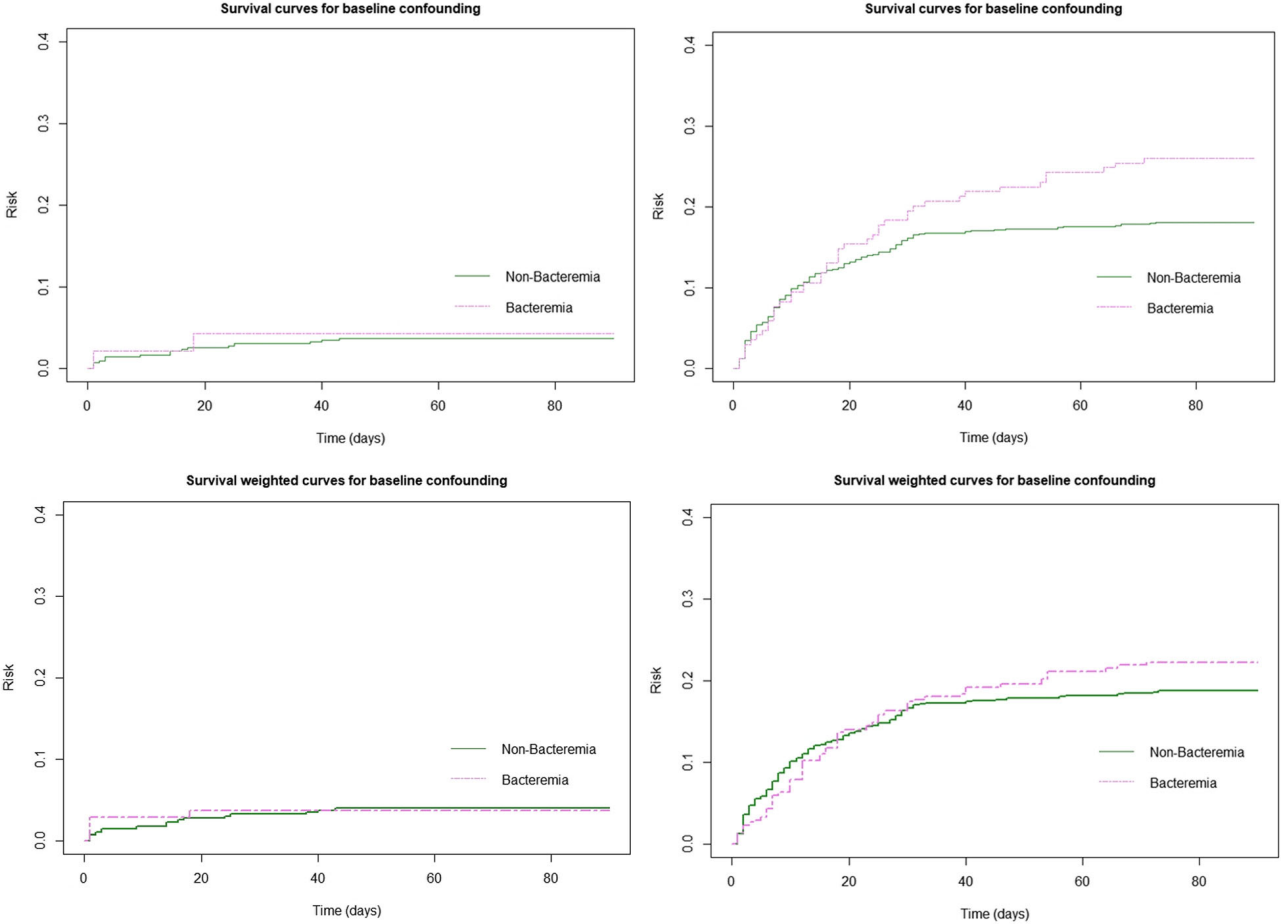
The 30-day Kaplan-Meier curves between patients with positive blood culture vs. negative blood culture in patients with sepsis (left panel) and severe sepsis or septic shock (right panel)

Given the difference of baseline covariates between culture-positive and culture-negative sepsis patients, we performed an IPTW (inverse probability of treatment weight) weighted analysis in the full cohort, the uncomplicated SIRS cohort, and the severe sepsis or septic shock cohort [21]. For early mortality, there was no significant differences between these three groups. For late mortality (30- to 90-day mortality), culture-positive sepsis was associated with increased odds of late mortality as compared with culture-negative sepsis in the full cohort (IPTW-OR, 1.95, 95%CI: 1.14–3.32) and in patients with severe sepsis or septic shock (IPTW-OR, 1.92, 95%CI: 1.10–3.33) (Table 3). To explore whether persistent Staphylococcal bacteremia is an important contributing factor to late mortality in culture-positive patients, we performed a sensitivity analysis by excluding patients with Staphylococcal bacteremia. After excluding Staphylococcal bacteremic patients, the risk of late mortality associated with culture-positivity attenuated and became not significant (IPTW-OR, 1.78, 95%CI: 0.87–3.62). We plotted the crude and IPTW-weighted Kaplan-Meier survival curve to facilitate visual comparison. (Fig. 1) In patients with severe sepsis and septic shock, there was no significant survival difference in the first 20 days, and the survival difference widened after the 20th day (Log-rank *P* <  0.001). In the IPTW-weighted survival curves, the survival difference narrowed, but the overall survival difference between culture-positive and culture-negative sepsis patients still remained significant, especially after day 30 (Log-rank *P* = 0.03).

**Table 3:** Comparison of survival difference between patients with culture-positive and culture-negative sepsis

	Subgroups	Crude OR Culture positive vs culture negative	IPTW-adjusted OR Culture positive vs culture negative
Early mortality	Full cohort	1.07 (0.64,1.80)	0.95 (0.56,1.62)
	SIRS	1.02 (0.13,8.23)	1.18 (0.19,7.31)
	Severe sepsis and septic shock	0.90 (0.521.53)	0.83 (0.47,1.44)
	Severe sepsis and septic shock, excluding Staphylococcal bacteremia	1.19 (0.65,2.19)	1.21 (0.65,2.26)
Late mortality	Full cohort	2.95 (1.81,4.79)***	1.95 (1.14,3.32)*
	SIRS	1.32 (0.16,10.94)	0.41 (0.02,11.25)
	Severe sepsis and septic shock	2.69 (1.62,4.48)***	1.92 (1.10,3.33)*
	Severe sepsis and septic shock,, excluding Staphylococcal bacteremia	2.4 (1.27,4.54)*	1.78 (0.87,3.62)

*p < 0.05, **p < 0.01, ***p <0.001

### Subgroup analysis

**Fig. 2:**
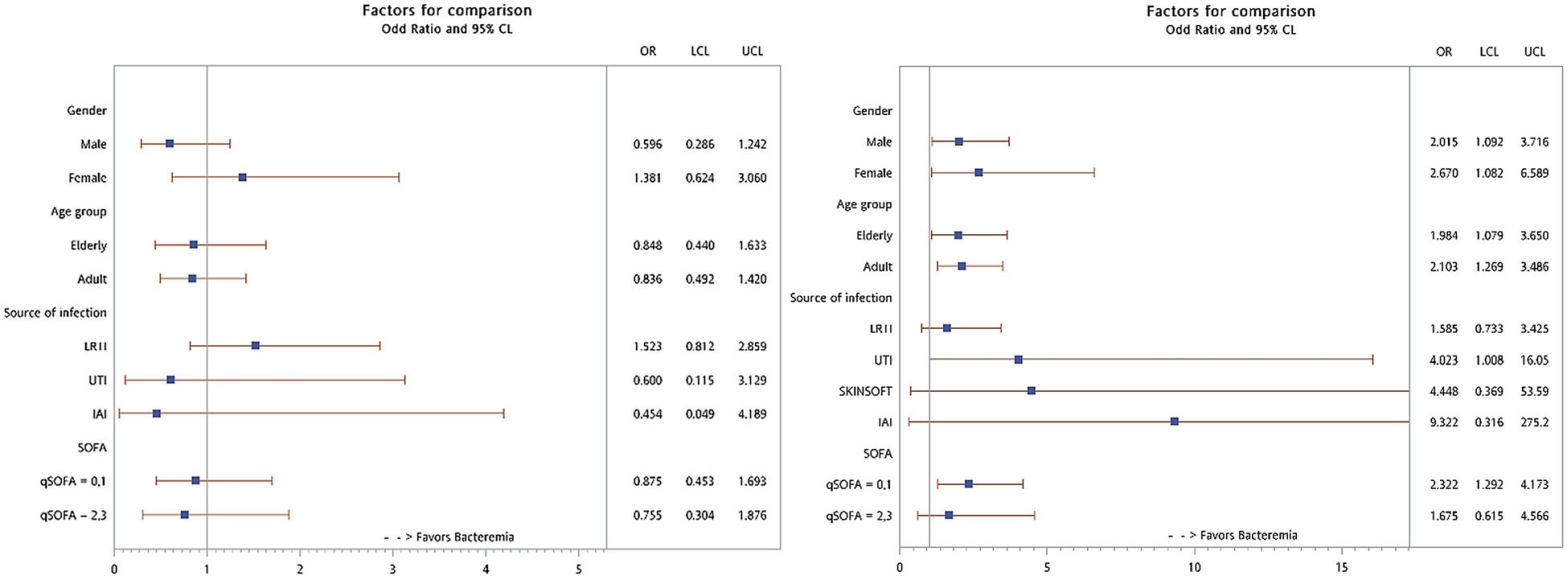
Odds ratio for early (30 days, left panel) and late (90 days, right panel) mortality between patients with blood culture positive and blood culture negative sepsis in different patient groups

We further performed subgroup analysis in several important clinical patient groups. There was no significant difference in early mortality between culture-positive patients and culture-negative patients across gender, age, source of infection, and clinical severity. For late mortality, we did not find that gender or age modified the culture-positive patients-associated mortality risk. However, we showed that culture-positive patients were associated with worse outcomes in more severe patients or in patients with UTIs (Fig. 2 and Supplementary Table 1).

## Discussions

In this multicenter cohort, we found that culture-positive sepsis patients have a higher comorbidity burden and higher clinical severity than culture-negative sepsis patients, but the 30-day mortality was comparable between the two groups. The culture-positive patients, however, had worse outcomes than the culture-negative patients between 31 and 90 days. The results were similar in patients with SIRS-defined sepsis, severe sepsis, or septic shock. Persistent staphylococcal bacteremia appeared to be a contributing factor to the increased risk of late mortality among patients with culture-positive sepsis.

Several studies have evaluated the impact of culture positivity on the outcome of sepsis patients without consistent events [2, 10, 11, 13]. In a prospective observational cohort study including 1001 medical ICU patients with severe sepsis, Phua J. et al. showed significant differences between culture-negative and culture-positive sepsis, including fewer comorbidities, milder severity of illness, shorter hospitalizations, and lower mortality for culture-negative patients [11]. However, the culture results status was not independently associated with mortality in multivariable analysis. Later, in a trinational retrospective cohort study (*n* = 8670), Kethireddy S. et al. showed that culture-negative septic shock patients experienced similar ICU survival (58.3% vs. 59.5%; *p* = 0.276) and overall hospital survival (47.3% vs. 47.1%; *p* = 0.976) to culture-positive septic shock patients [2]. Additionally, Sigakis M.J.G. et al. utilized 8 years of patient records in a large academic medical center comprising 9288 culture-negative patients (89%) and 1105 culture-positive patients (11%) to show that a positive culture was not independently associated with mortality in sepsis defined by SIRS (OR: 1.01, 95% CI, 0.81–1.26), sequential organ failure assessment (SOFA) score (OR: 1.13, 95% CI, 0.86–1.43), and quick SOFA score (OR: 1.05, 95% CI, 0.83–1.33), respectively [13]. While most of these studies found no difference in survival between culture-positive and culture-negative patients, Gupta, S. et al. conducted a large cohort study using the Nationwide Inpatient Sample (NIS) comprising 6,843,279 admissions of patients with severe sepsis and showed that 47.1% of patients had culture-negative results [10]. Those with culture-negative severe sepsis had more comorbidities and acute organ dysfunction (respiratory, cardiac, hepatic, and renal dysfunction), and culture-negative severe sepsis was independently associated with in-hospital mortality (OR, 1.75; 95% CI, 1.72–1.77). Our study corroborates with Phua J. et al’s finding that patients with culture-positive sepsis have higher severity and mortality than those with culture-negative sepsis; however, there was no difference in 30-day mortality between the two groups of patients after IPTW weighting [11]. Between 31 to 90 days, culture-positive sepsis has a significantly higher mortality than culture-negative sepsis.

None of the previous studies reported the survival curves between two groups of patients, so we could not compare our late mortality difference results with those of previous studies. We suspect that persistent *Staphylococcus aureus* bacteremia (SAB) is an important contributing factor to the increased late mortality of culture-positive sepsis patients as compared with culture-negative sepsis patients. Excluding patients with documented SAB attenuated the late mortality risk of culture-positive patients and narrowed the survival difference between culture-positive and culture-negative patients to a statistically nonsignificant status. Persistent SAB, described as SAB that persists for 3–7 days, is a well-documented problem [22, 23]. Persistent bacteremia causes high rates of morbidity and attributable mortality [24]. Reported independent risk factors for persistent bacteremia included community-onset bacteremia, bone and joint infection, central venous catheter-related infection, metastatic infection, suppurative thrombophlebitis, infective endocarditis, and methicillin resistance [2, 23]. It has been shown that bacteremia may persist for ≥7 days even after removal of CVC, so early source control and higher vancomycin trough levels should be implemented [25]. Duration of antibiotic treatment has been shown to be a key factor to the survival of patients with persistent SAB. Abbas M. et al. evaluated the effect of duration of therapy (DOT) on mortality and relapse in patients with SAB. In a retrospective, single-center cohort study including 225 patients with uncomplicated SAB and 305 patients with complicated SAB. After carefully accounting for confounding and immortal time bias, they concluded that in patients with complicated SAB, DOT > 14 days was associated with a higher survival rate than DOT < 14 days. Other causes can explain the comparable mortality of culture-negative sepsis patients with culture-positive sepsis patients despite the more severe clinical presentations.

In total, 18 patients were excluded from final analysis, of which 7 were excluded due to transferring to other hospitals during the follow-up period, 6 lost follow-up, and the remaining was excluded due to missing data. The outcome information of patients transferred to other hospitals or lost to follow-up was not available. However, we recorded the reasons for transferal. As the three participating hospitals are the largest medical centers locally, the reasons for transferring to other hospitals were the geographic proximity of caregivers rather than the deterioration of clinical conditions. Therefore, these patients lost to follow-up are highly unlikely to have the worst outcome to bias our analysis. On the contrary, including these patients lost to follow-up may even improve the overall outcome.

### Strengths and limitations

The results of this study should be interpreted in light of its strengths and weaknesses. The multi-centered Asian patient cohort that included patients from intensive care units, emergency departments, and hospital wards increases the generalizability and clinical applicability of the study in Asian populations. The use of an IPTW approach instead of PS matching or traditional regression increases the statistical efficiency and allows the estimation of population average effect. The IPTW-weighted survival curve enables the survival differences of the two comparison groups to be visualized over time, allowing the time-dependent impact of culture positivity on different stages of sepsis to be identified. There are, of course, limitations in our study. First, because of the observational character of the study, there is always a possibility for unmeasured confounding factors. Second, information on prior exposure to antibiotics before hospital admission, an important determinant of culture positivity, was lacking. The outcome impact of culture positivity may also involve the possible increased use of prehospital antibiotics in the culture-negative patients. Third, unlike previous studies that included all types of microbiological culture results, we only focused on the initial blood culture results. Gupta, S. et al.’s study, which utilized an administrative database, was the only other study that showed increased mortality associated with culture-negative sepsis [10]. In that study, culture positivity was defined as the presence of any ICD-9 code for all kinds of microbiology diagnoses. It is likely that the immortal time bias played a role in their exceptional findings, as patients with longer length of hospital stay tend to receive more microbiological tests and hence more microbiological diagnoses. The immortal time bias was not amenable to regression analysis. Most of the positive blood culture results (97.7%) of our study came from the initial blood cultures. Therefore, the risk of immortal time bias in this cohort is minimal.

## Conclusions

After adjusting for potential confounders, this study shows that culture positivity does not have a significant impact on 30-day mortality. These results were robust in patients with severe sepsis or septic shock. Nevertheless, we found that culture-positive patients may have worse outcomes than culture-negative patients after 30 days. Persistent staphylococcal bacteremia may be a contributing factor to increased late mortality among patients with culture-positive sepsis. These findings are worth further attention.

## Supplementary Information

**Additional file 1 **: **Supplementary Table 1**. Subgroup analysis for risk of early or late mortality between patients with culture positive and blood culture negative sepsis. **Supplementary Table 2**. Model of propensity score (AUC: 0.68). **Supplementary Table 3**. Microbiology of bacteria isolated. **Supplementary Figure 1**. Patient inclusion flowchart. **Supplementary Figure 2**. Covariate balance before (blue) and after (red) IPTW weighting for culture negative versus culture positive sepsis patients. Improved balance (i.e., reduced standardized differences) was obtained after IPTW weighting.

## Data Availability

The data sets generated and/or analyzed during the current study are not publicly available due to the data confidentiality requirements of the ethics committees, but are available from the corresponding author on reasonable request and approval from the ethics committees in all institutions.

## Abbreviations

IPTW: Inverse probability of treatment weighting
ICU: Intensive care unit
SIRS: Systemic inflammatory response syndrome
DNR: Do-not-resuscitate
SOFA: Sequential organ failure assessment
GCS: Glasgow coma score
BSI: Bloodstream infection
PS: Propensity score
LRTI: Lower respiratory tract infection
MEDS: Mortality in Emergency Department Sepsis
NIS: Nationwide Inpatient Sample
SAB: *Staphylococcus aureus* bacteremia
