# Supplementary material for: Comparing mortality between positive and negative blood culture results: an inverse probability of treatment weighting analysis of a multicenter cohort
Source: BMC Infect Dis. 2021 Feb 17;21:182. doi: 10.1186/s12879-021-05862-w (PMC7887786; doi:10.1186/s12879-021-05862-w)

Supplementary Table 1. Subgroup analysis for risk of early or late mortality between patients with culture positive and blood culture negative sepsis.

	**Early mortality**		**Late mortality**	
	**OR(95% CI)**	**P value**	**OR(95% CI)**	**P value**
**Gender**				
Male	0.6(0.29,1.24)	0.167	2.02(1.09,3.72)	0.025*
**Age group**				
Elderly (>=65 year old)	0.85(0.44,1.63)	0.622	1.98(1.08,3.65)	0.028*
Adult (>=18 year old)	0.84(0.49,1.42)	0.507	2.1(1.27,3.49)	0.004**
**Source of infection**				
Lower respiratory tract infection	1.52(0.81,2.86)	0.190	1.59(0.73,3.43)	0.242
Urinary tract infection	0.6(0.12,3.13)	0.544	4.02(1.01,16.05)	0.049*
Skin and musculoskeletal infection	-	0.967	4.45(0.37,53.59)	0.240
Intra-abdominal infection	0.45(0.05,4.19)	0.486	9.32(0.32,275.24)	0.196
**SOFA**				
qSOFA = 0,1	0.88(0.45,1.69)	0.692	1.68(0.62,4.57)	0.313
qSOFA = 2,3	0.76(0.3,1.88)	0.545	2.32(1.29,4.17)	0.005**

***** $p<0.05$, ** $p<0.01$

Supplementary Table 2. Model of propensity score (AUC: 0.68)

	OR (95%CI)	P value
**Demographics**		
Male sex	1.14(0.83,1.58)	0.418
Age	1.04(0.98,1.10)	0.240
Age squared	1.00(1.00,1.00)	0.453
Nursing home residents	1.09(0.58,2.05)	0.796
**Comorbidities**		
Diabetes mellitus	1.70(1.21,2.37)	0.002**
COPD	1.22(0.67,2.21)	0.512
Stroke	0.97(0.62,1.51)	0.889
Myocardial infarction	0.64(0.27,1.53)	0.313
Congestive heart failure	1.24(0.73,2.12)	0.421
Chronic kidney disease	1.24(0.76,2.02)	0.384
Chronic liver disease	1.25(0.78,2.02)	0.354
Malignancies	1.38(0.89,2.16)	0.155
**Site of primary infection**		
Lower respiratory tract infection	0.79(0.57,1.08)	0.142
Urinary tract infection	1.85(1.20,2.85)	0.005**
Skin and musculoskeletal infection	1.25(0.55,2.85)	0.601
Hepatobiliary infection	2.45(1.39,4.32)	0.002**
Intra-abdomen infection	0.98(0.53,1.81)	0.946
**Organ dysfunction**		
SOFA score	1.03(0.91,1.17)	0.633
SOFA score squared	1.00(0.99,1.01)	0.506

* p<0.05, ** p<0.01

Supplementary Table 3. Microbiology of bacteria isolated

Microbiology of bacteria isolated	Number (%)
Escherichia coli	53 (24.5%)
Klebsiella pneumoniae	21 (9.7%)
Staphylococcus epidermidis	20 (9.3%)
Staphylococcus aureus	14 (6.5%)
Staphylococcus hominis	10 (4.6%)
Coagulase neg. staphylococci	10 (4.6%)
Staphylococcus haemolyticus	8 (3.7%)
Streptococcus agalactiae	3 (1.4%)
Pseudomonas aeruginosa	3 (1.4%)
Penicillium marneffei	3 (1.4%)
Enterococcus faecium	3 (1.4%)
Enterobacter cloacae	3 (1.4%)
Acinetobacter baumannii	3 (1.4%)
Viridans streptococci	2 (0.9%)
Salmonella	2 (0.9%)
Other gram negative bacilli	2 (0.9%)
Other enterococcus species	2 (0.9%)
Enterococcus faecium	2 (0.9%)
Streptococcus pneumoniae	1 (0.5%)
Streptococcus mitis	1 (0.5%)
Streptococcus intermedius	1 (0.5%)
Streptococcus anginosus	1 (0.5%)
Stenotrophomonas maltophilia	1 (0.5%)
Staphylococcus xylosus	1 (0.5%)
Staphylococcus warmeri	1 (0.5%)

Staphylococcus cohnii	1 (0.5%)
Staphylococcus capitis	1 (0.5%)
Sphingomonas paucimobilis	1 (0.5%)
Ochrobactrum anthropi	1 (0.5%)
Morganella morganii	1 (0.5%)
Moraxella species	1 (0.5%)
Micrococcus luteus	1 (0.5%)
Klebsiella oxytoca	1 (0.5%)
Fusobacterium necrophorum	1 (0.5%)
Enterococcus faecalis	1 (0.5%)
Capnocytophaga sputigena	1 (0.5%)
Candida glabrata	1 (0.5%)
Brucella melitensis	1 (0.5%)
Aeromonas hydrophila	1 (0.5%)
Polymicrobial infection	31(14.4%)

Supplementary Figure 1. Patient inclusion flowchart

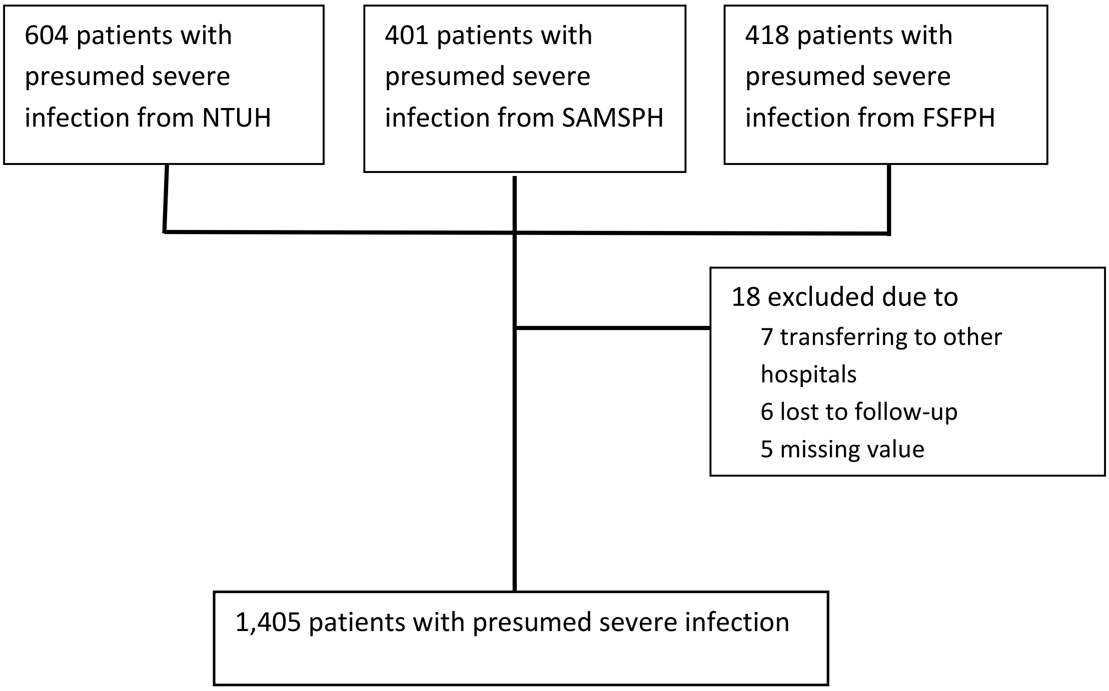

NTUH: National Taiwan University Hospital; SAMSPH: Sichuan Academy of Medical Sciences and Sichuan Provincial People’s Hospital; FSFPH: The First People's Hospital of Foshan; ICU: intensive care unit

Supplementary Figure 2. Covariate balance before (blue) and after (red) IPTW weighting for culture negative versus culture positive sepsis patients. Improved balance (i.e., reduced standardized differences) was obtained after IPTW weighting.

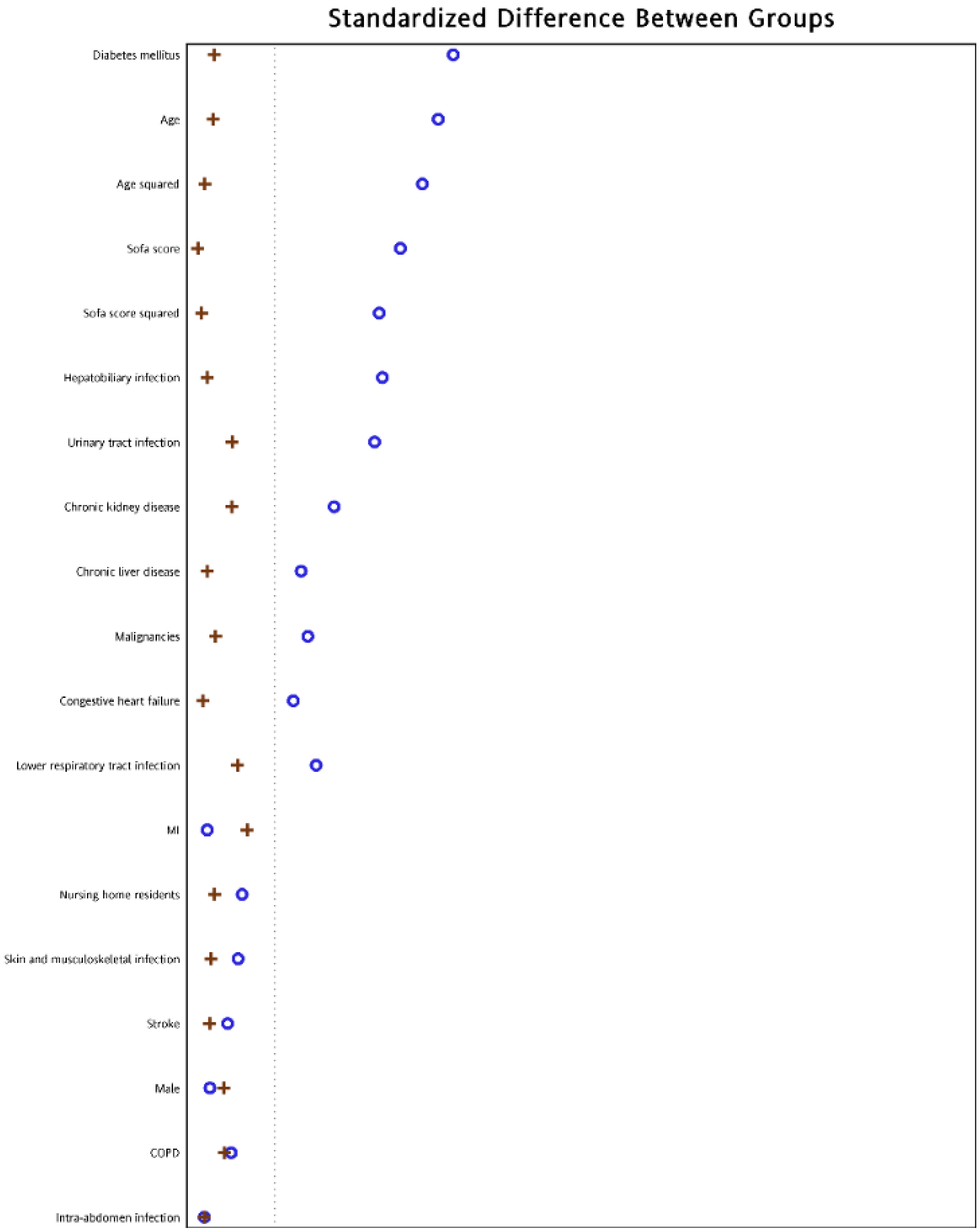

Supplement: Supplementary file 1 — Additional file 1 : Supplementary Table 1. Subgroup analysis for risk of early or late mortality between patients with culture positive and blood culture negative sepsis. Supplementary Table 2. Model of propensity score (AUC: 0.68). Supplementary Table 3. Microbiology of bacteria isolated. Supplementary Figure 1. Patient inclusion flowchart. Supplementary Figure 2. Covariate balance before (blue) and after (red) IPTW weighting for culture negative versus culture positive sepsis patients. Improved balance (i.e., reduced standardized differences) was obtained after IPTW weighting. [file 12879_2021_5862_MOESM1_ESM.pdf]
